# Dr. Thomas H. Chandler

**Published:** 1895-12

**Authors:** 


### Obituary.

### DR. THOMAS H. CHANDLER.

Died, at his residence, 72 St. Stephen street, Boston, Mass., on Tuesday, August 27, 1895, THOMAS HENDERSON CHANDLER, A. M., LL. B., M. D., D. M. D., dean and professor of mechanical dentistry in the dental department of Harvard University, aged seventy-one years, one month and three days.

Dr. Chandler was the oldest of a family of five brothers. Born in the old Chandler homestead at the North End, Boston, he brought himself by hard and untiring work to a prominent position among the literary and professional men of that city. His early education was obtained in the old Eliot School from which he was graduated at the head of his class, with the Franklin medal. He next entered the Boston Latin School, under Master Dixwell, graduating four years later as Franklin medalist and class leader. Havard's doors now stood open to him, and he entered, after passing the admission examination with honors. His college career was as successful as had been his previous scholastic efforts, and he obtained several prizes for excellence in his studies, and graduated as a Phi Beta Kappa man and president of the Hasty Pudding Club. Having decided to follow the profession of the law, he entered the Harvard Law School, graduating in the class of 1853.

His eyes now commencing to trouble him, he applied for thn post of usher in the Boston Latin School, and obtained it through his high scholarship and testimonials to his capabilities given him by Edward Everett, president of Havard College. In 1858 he began the study of dentistry, a science then almost in its infancy, and some years later studied medicine, retaining, however, dentistry as his specialty. On the organization of the Harvard Dental School in 1869, he was offered the post of adjunct professor of mechanical dentistry, and, on the resignation of Dr. N. C. Keep in 1872, he was appointed professor with the degree of D. M. D. *honoris causa.*

The death of Dr. Thomas B. Hitchcock in 1874 left the school without a dean, and Dr. Chandler was unanimously elected to fill the vacancy. During the twenty-one years he held this post, never until the few months preceding his death did he fail in attending a single meeting of the faculty or miss fulfilling a single duty connected with the office. The students all liked him, and his associates all respected him, for he was a man of the most unobstrusive and retiring disposition, which was often carried to the pitch of diffidence.

His literary work consisted chiefly of papers for the different medical and dental journals; an exhaustive article on

"Thumb-sucking in Childhood and its Results" was translated into several European languages, and obtained a European reputation for its author. Translations of two works on dental caries, one by Leber and Rottenstein, and the other from the French of Magitot, were the chief events of his literary career. In this epoch of push and hurry it can be said of few persons that their work was done slowly, thoroughly, and to last. Dr. Chandler's ambition was not to be a shining light, but to use his best judgment, his utmost skill, and the greatest care in every task, however trivial, that he undertook. He was a good husband, a kind father, a true citizen, and an honest man.—*Dental Cosmos.*

